# Invasive non-typhoidal *Salmonella* infections in China (1961–2024): a retrospective systematic analysis of multicentre case reports

**DOI:** 10.7189/jogh.16.04008

**Published:** 2026-01-12

**Authors:** Haiyang Zhou, Chenghao Jia, Qianzhe Cao, Linlin Huang, Lin Teng, Zining Wang, Chenghu Huang, Fang He, Yan Li, Guoping Zhao, Min Yue

**Affiliations:** 1 Department of Veterinary Medicine, College of Animal Sciences, Zhejiang University, Hangzhou, China; 2 Hainan Institute of Zhejiang University, Hangzhou, China; 3 School of Life Science, Hangzhou Institute for Advanced Study, University of Chinese Academy of Sciences, Hangzhou, China

## Abstract

**Background:**

*Salmonella* can be classified as either typhoidal or non-typhoidal. The former primarily causes invasive infections, while the latter typically results in self-limiting diarrhoea. Infections caused by invasive non-typhoidal *Salmonella* (iNTS) are becoming an emerging global health burden, particularly in low- and middle-income regions. While they account for thousands of deaths each year, we still lack systematic analysis on their burden and epidemiology in Asia, particularly in China.

**Methods:**

We searched Web of Science, PubMed, China National Knowledge Infrastructure, Wanfang Data, Airiti Library, and the China Science and Technology Journal Database on 31 March 2024. The primary outcomes of interest included the spatiotemporal distribution of iNTS infection cases, patients’ characteristics, and clinical outcomes. The secondary outcomes encompassed characterisation of isolates, medical histories, and medication usage. We used the Open Meta-Analyst software to estimate the case fatality rate.

**Results:**

We included 199 publications for analysis. Eastern regions of China were identified as hotspots for infection, males and children were especially susceptible populations, while trauma and metabolic diseases emerged as major risk factors associated with infection. Typhimurium, Choleraesuis, and Enteritidis were the top three serovars responsible for infections, with the bloodstream being the most frequent site of invasion. The overall case-fatality rate of iNTS in China was 8.6% (95% confidence interval = 6.8–10.4).

**Conclusions:**

In exploring the epidemiological trends of iNTS infections in China, we observed significant risk associations with both patient characteristics and pathogen-specific determinants. There is an urgent need to establish enhanced surveillance systems and implement geographically tailored interventions+, particularly in economically underdeveloped regions.

**Registration:**

PROSPERO (CRD42024569499).

Bacterial infections are the second leading cause of death worldwide, resulting in approximately 7.7 million deaths annually [1]. In China, the rise of invasive infections caused by Gram-negative bacteria remains a significant public health concern [2–6]. *Salmonella*, a common pathogen with >2600 serovars, significantly contributes to this burden. Based on the patient’s clinical symptoms, *Salmonella* infections are often categorised as either typhoidal or non-typhoidal, with the former characterised by invasive high fever, and the latter typically causing self-limited diarrhoea [7–14]. In recent years, non-typhoidal *Salmonella* has emerged as a significant cause of extra-intestinal bacterial infections in certain low- and middle-income countries [15–18], including China, marking a major shift in the epidemiological landscape [19]. It is estimated that, globally, invasive non-typhoidal *Salmonella* (iNTS) alone causes ~ 77 500 deaths annually [20–22]. Most of these patients exhibit underlying conditions or immunosuppression, such as metabolic diseases, HIV/AIDS, cancer, and autoimmune diseases [23–25]. While recent studies have shown that non-typhoidal *Salmonella* has been becoming more invasive in regions already heavily affected by iNTS, particularly sub-Saharan Africa [26–28], we still lack data for most other parts of the world [29–32].

The World Health Organization (WHO) has included investigating invasive bacterial infections in low- and middle-income countries in the Global Research Agenda for Antimicrobial Resistance in Human Health programme [33]. However, to date, most systematic investigations of iNTS infections have focused exclusively on Africa, leaving many blind spots regarding global epidemiological trends and risks. China, for example, carries a major burden of foodborne disease outbreaks, with *Salmonella* playing a significant role in this sense [34–38]. While the number of cases caused by typhoid *Salmonella* in the country has been declining in recent years [39], we lack detailed and accurate investigations into invasive diseases caused by iNTS [40–42], creating obstacles for adequate treatment and prevention, including the formulation of preventive policies [43,44]. There has been little research in this context, with studies mostly reporting on sporadic cases, preventing us from fully understanding the epidemiology of iNTS in China, particularly regarding predominant serovars, susceptibility for infection, treatment regimens, and clinical outcomes.

To address this gap, we systematically reviewed public data related to iNTS infection to better understand its occurrence, temporal and regional distribution, antimicrobial resistance patterns, and disease burden in China.

## METHODS

We preregistered our study on PROSPERO (CRD42024569499) and reported it per the PRISMA [45] and STROBE statements [46].

### Search strategy

We searched Web of Science, PubMed, the China National Knowledge Infrastructure, Wanfang Data, Airiti Library, and the China Science and Technology Journal Database for English- or Chinese-language literature on iNTS. The search strings for Web of Science and PubMed comprised targeted keywords such as ‘Salmonella’ and ‘China’ to provide a geographical focus, while the strategy for the Chinese databases contained no geographic restrictions, as most Chinese studies report on domestic populations (Table S1 in the **Online Supplementary Document**). We conducted the literature search on 31 March 2024, without any temporal restrictions.

### Inclusion and exclusion criteria

We employed a two-stage screening strategy approach (Figure S1 in the **Online Supplementary Document**). 

In the first stage, six reviewers independently screened the titles and abstracts to exclude studies lacking epidemiological or clinical information, such as laboratory-based research focusing solely on the biological mechanisms of *Salmonella*. In the second stage, three reviewers performed full-text screening for all pre-selected articles, excluding studies that reported on *Salmonella* infections confined to intestinal sites or studies that did not report specific non-typhoidal serovars. Discrepancies between reviewers were resolved through discussion. Based on the two-stage screening, 354 pre-selected articles underwent full-text screening. We performed the screening in the Rayyan software (Rayyan, Cambridge, Massachusetts, USA).

### Data extraction and quality assessment

The extracted publication information included authors' names, article titles, journal names, volume numbers, issue numbers, publication years (categorised them into four time periods: ≤1989, 1990–99, 2000–09, and 2010–24), and DOIs. To ensure traceability, we further recorded translations of Chinese-language publications and the primary institutional affiliation of the first author. Hospitals within the studies were categorised into tertiary, secondary, and primary institutions following China’s official healthcare tiering system (Table S2 in the **Online Supplementary Document**).

#### Epidemic data

Epidemic data primarily includes information on the origin of the infection, *i.e.* the time and location of its occurrence. Notably, most case reports do not specify the exact location of exposure; therefore, since cross-regional consultations are not frequent, especially at the provincial administrative level, we approximated the hospital address where the patient sought treatment as the location of disease onset. Economic regions are referenced from official documents from the National Bureau of Statistics of China, which reflect variations in socioeconomic development and public health resource allocation across provinces. Specifically, the Northeastern region includes Liaoning, Jilin, and Heilongjiang; the Eastern region comprises Beijing, Tianjin, Hebei, Shanghai, Jiangsu, Zhejiang, Fujian, Shandong, Guangdong, Hainan, Taiwan, Hong Kong Special Administrative Region, and Macau Special Administrative Region; the Central region includes Shanxi, Anhui, Jiangxi, Henan, Hubei, and Hunan; and the Western region covers Inner Mongolia, Guangxi, Chongqing, Sichuan, Guizhou, Yunnan, Tibet, Shaanxi, Gansu, Qinghai, Ningxia, and Xinjiang.

#### Case cohort

The case cohort included the number of patients reported, gender ratio, age, and specific sites of extraintestinal iNTS infection, and comorbidities such as diabetes, AIDS, autoimmune diseases, digestive system disorders, trauma, and cancer.

#### Pathogen data

Pathogen data covers the specific serovars, serogroups, and clinical identification methods used for microbiologically confirmed iNTS cases. In some instances, the same isolate was validated using multiple laboratory methods.

#### Treatment data

Treatment information includes data on the medications and other therapeutic methods, and the patients’ clinical outcomes. Antimicrobials were recorded using abbreviations (Table S3 in the **Online Supplementary Document**). Some patients did not achieve clinical improvement through antibiotic treatment alone; their symptoms improved after surgical intervention. These cases are also documented in the dataset. We further extracted antimicrobial susceptibility data, when available, and summarised susceptibility patterns using a descriptive frequency-based approach. For each antimicrobial, we recorded how many times isolates were reported as ‘susceptible’ or ‘resistant’, providing a pragmatic overview of historical susceptibility trends and enabling readers to gauge the potential clinical relevance of each drug.

### Statistical analysis

We used descriptive statistics to summarise the characteristics of iNTS cases, presenting categorical variables (*e.g.* sex, serovars, underlying conditions, treatment approaches) as frequencies and percentages. We also descriptively summarised the temporal and geographical distributions of reported cases. All descriptive analyses were conducted using Microsoft Excel (Microsoft Corp., Redmond, Washington, USA).

We estimated the case fatality rate using the Open Meta-Analyst software (Agency for Healthcare Research and Quality, Rockville, Maryland, USA), employing the binary random-effects model with a 95.0% confidence level, as detailed in other studies [47–52]. For geographic region and sex, we calculated a risk-of-death index, defined as the ratio of the subgroup’s share of total deaths to its share of total reported cases and calculated per the formula: risk of death = (subgroup deaths/total deaths)/(subgroup cases/total cases)

## RESULTS

### Data integration and summary

Our search retrieved 8264 records, with 1436 removed through deduplication and 6828 retained for screening. We selected 199 publications related to iNTS from the initial 8264 articles, extracting 576 recorded cases of iNTS infection in total, as well as epidemiological data, patient cohorts, pathogen information, and treatment processes. Nine publications were published before 1989, and 79 in the 2000–09 period **(**Figure S1 in the **Online Supplementary Document**). The studies reported on 576 cases of iNTS infections distributed consistently across the study period, with the fewest occurring in 2010–24 period (n = 112) and the most in 2000–09 (n = 168).

### Spatiotemporal trends

The 576 cases accounted for most of the provincial-level administrative regions in China, except Hainan Province and the Tibet Autonomous Region, where no data were available. The majority of reported cases were concentrated in the central and eastern economic zones of China. Sichuan and Ningxia had high case reports within the western economic zone (Figure S2 in the **Online Supplementary Document**). However, when considering the temporal scale, the central and eastern economic zones exhibited different development patterns. The number of reported cases in the central economic zone has steadily declined over the years, while we noted an inverse trend in the eastern economic zone. Furthermore, most cases (over 70%) were reported from tertiary grade A hospitals, which are typically comprehensive academic medical centres located in urban or provincial capitals. This pattern may partly reflect referral pathways toward higher-level hospitals and the greater likelihood that cases managed in tertiary, high-level institutions are reported and published (Figure S3 in the **Online Supplementary Document****)**.

### Meta-analysis of patient information

**Figure 1:**
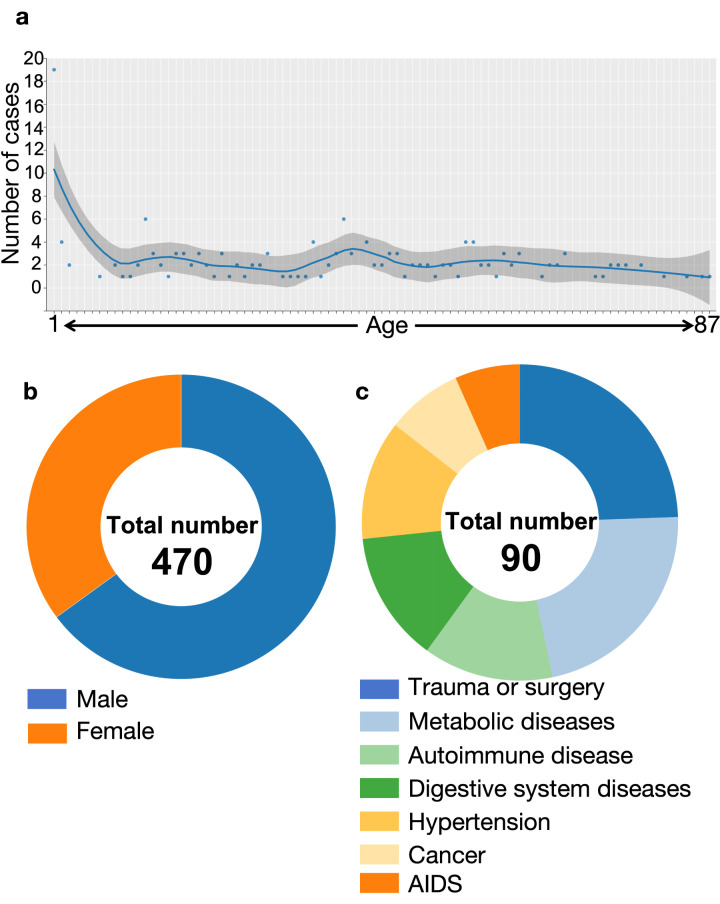
Meta-analysis of patient information. **Panel A.** Age distribution chart of patients, with a total of 165 cases reporting the patient's age. The maximum age is 87 years. The horizontal axis represents age, and the vertical axis represents the number of patients. **Panel B.** Pie chart of the gender ratio of patients. **Panel C.** The proportion of other comorbidities among patients.

The average age of the patients with available age data (n = 165) was 35.5 years, with a significantly higher number of newborn patients aged 0–1 compared to other age groups (**Figure 1**, Panel A). Of the 470 cases with recorded genders, approximately 65% were male, while about 35% were female (**Figure 1**, Panel B). Aside from the iNTS infection, information on comorbidities was available for 90 cases, with ~24% patients reporting a history of trauma or surgery; ~22% having metabolic disorders (such as diabetes or gout), ~13% having autoimmune diseases (such as lupus); ~13% having digestive system diseases (mainly liver or gallbladder conditions); ~12% suffering from hypertension, ~ 7% having cancer, and ~ 6% being HIV-positive (**Figure 1**, Panel C).

### Aetiological characteristics of iNTS infections

Serotyping was performed on all isolated strains, identifying a total of 46 serovars. The top five serovars were Typhimurium (n = 277, 48.09%), Choleraesuis (n = 124, 21.53%), Enteritidis (n = 40, 6.94%), Dublin (n = 38, 6.60%), and Newport (n = 8, 1.39%) (Figure S4 in the **Online Supplementary Document**). iNTS infections caused by Typhimurium accounted for the vast majority of 150 cases occurring before the year 1989 (n = 143, 95.33%). This proportion decreased steadily over the following 20 years, but has recently shown a resurgence, with a significant rise in reports of the serovar Choleraesuis during 2000–09 (n/N = 99/168, 58.93%), making it the primary pathogenic factor of that period. *Salmonella* was isolated from multiple specimen types in 97 of the 576 cases. Among the 479 cases where *Salmonella* was isolated from a single culture, blood was the most common source (n = 390, 81.92%), followed by pus (n = 29, 6.05%), cerebrospinal fluid (n = 19, 3.97%), synovial fluid (n = 16, 3.34%), and bile (n = 6, 1.25%).

### Mortality rates and risk of death index

**Figure 2:**
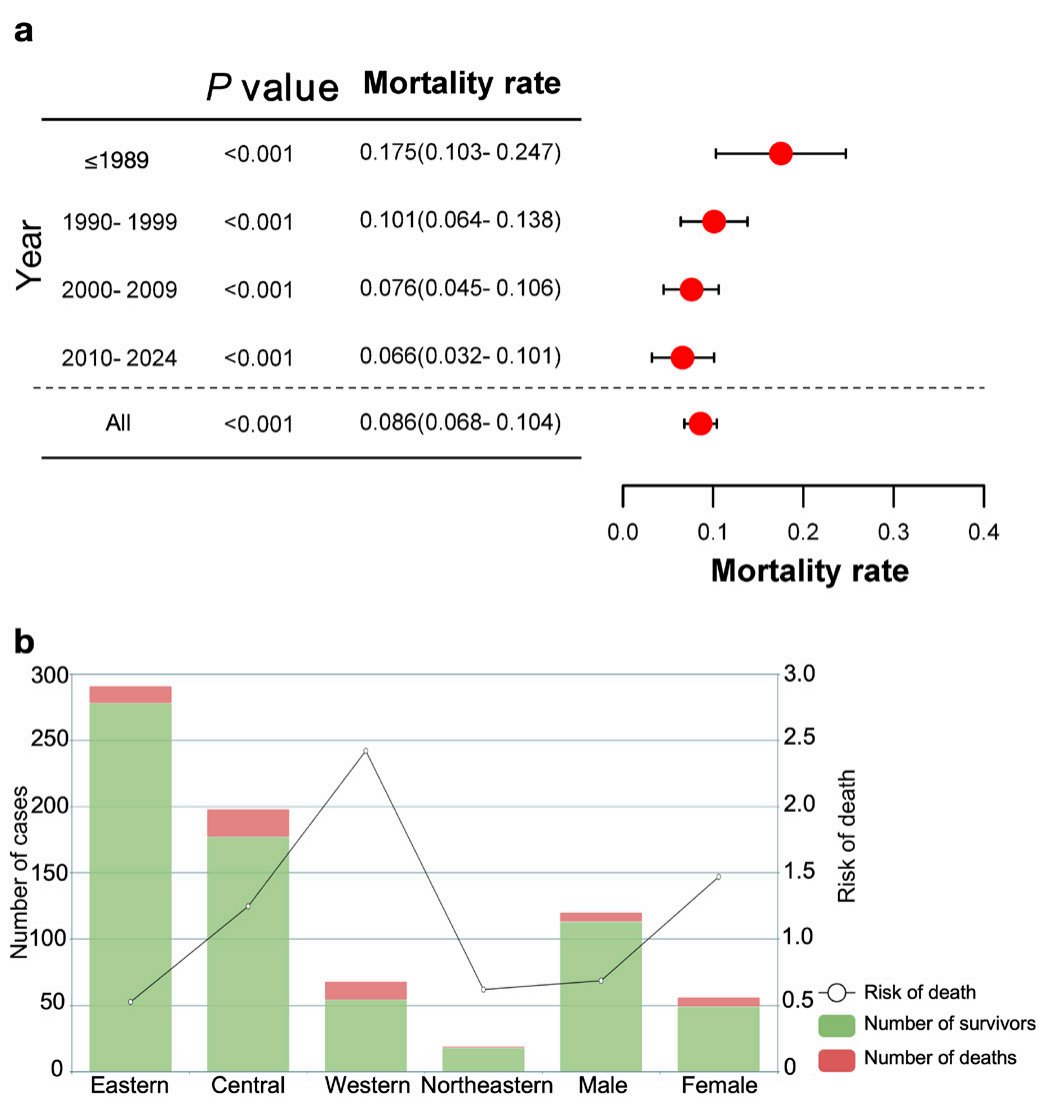
Mortality rates and risk of death under different factors. **Panel A.** The dashed regions represent the case mortality rates for various time periods and the overall population. **Panel B.** The risk of death and the distribution of patients with different clinical outcomes under different factors.

Among the 576 cases, 49 had a clinical outcome of death, with an estimated mortality rate of 8.6% (95% confidence interval (CI) = 6.8–10.4). The mortality rate of iNTS infections gradually decreased over time, from 17.5% (95% CI = 10.3–24.7) in the pre-1989 period to 6.6% (95% CI = 3.2–10.1) in 2010–24 (**Figure 2**, Panel A). As described above, we ran subgroup analyses to evaluate the influence of geographic region and sex on the risk of death. Although the Eastern region accounted for approximately half of all reported cases, it contributed only 25.6% of total deaths, with the lowest risk index of 0.53 among the four regions. In contrast, the Northeastern, Central, and Western regions had risk of death indices of 0.62, 1.25, and 2.42, respectively. Male patients had a markedly lower risk of death index (0.69) compared with female patients (1.47) (**Figure 2**, Panel B).

### Antimicrobial resistance and clinical characteristics

**Figure 3:**
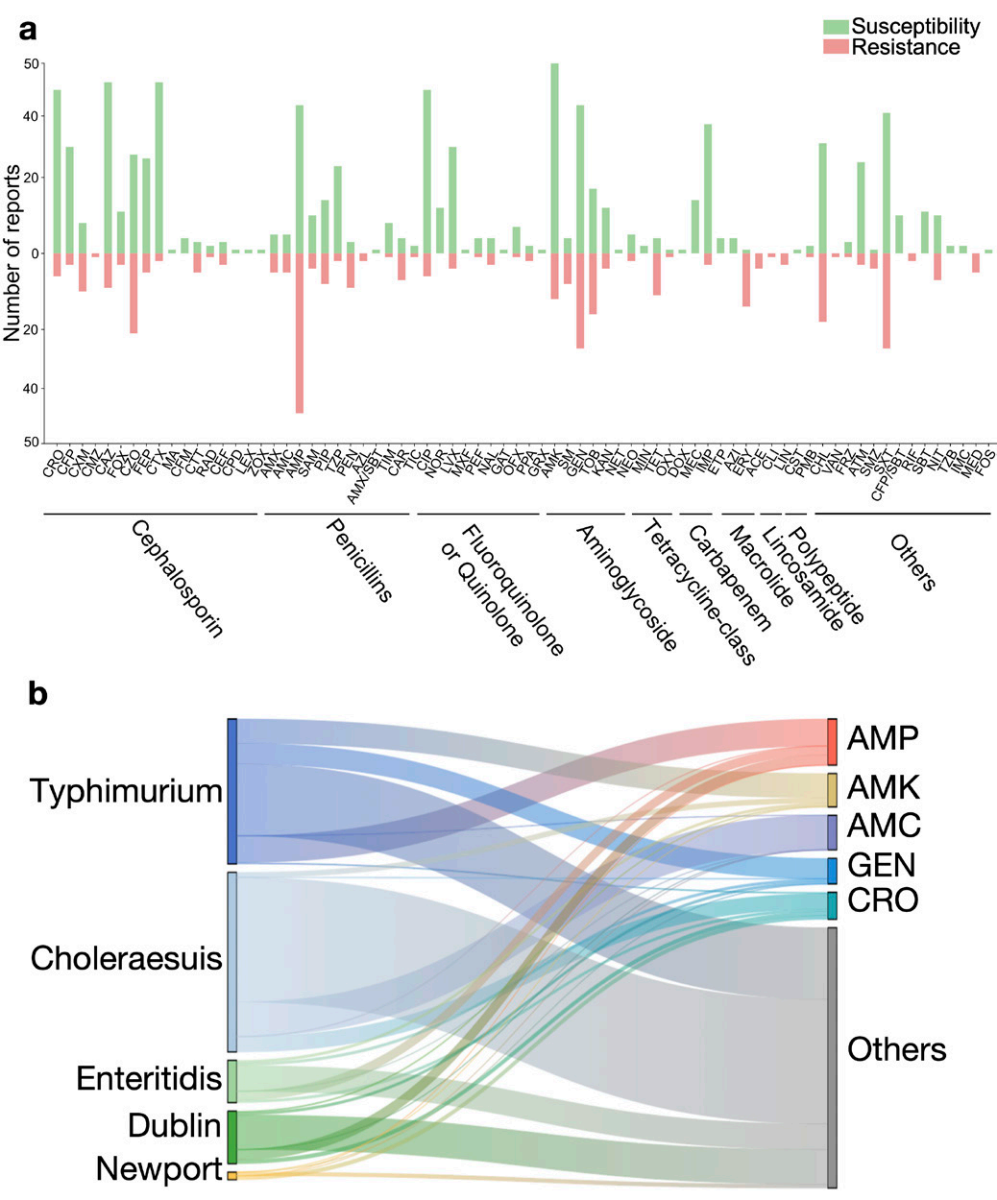
Clinical characteristics among different *Salmonella* serovars. **Panel A.** Number of reports describing susceptibility and resistance to different antimicrobials across all included studies. Green bars represent the number of times an antibiotic was reported as susceptible or intermediate, and red bars represent the number of times it was reported as resistant. The full names and classifications of antimicrobials abbreviations are provided in Table S3 in the **Online Supplementary Document**. **Panel B.** The correspondence between the top five serovars and the top five antimicrobials. AMC – amoxicillin and clavulanate potassium, AMK – amikacin, AMP – ampicillin, CRO – ceftriaxone, GEN – gentamicin.

Of the 199 included studies, 117 reported on antimicrobial susceptibility testing results, encompassing a total of 74 distinct agents. For each antimicrobial, we aggregated and reported the number of times it was described as ‘susceptible’ or ‘resistant’. Cephalosporins were the most frequently tested drug class, with most agents showing a substantially higher frequency of susceptibility reports compared to resistance, except cefuroxime, cefmetazole, cefotetan, and cephalothin. Several other antimicrobials (*e.g.*, TZP, CIP, NOR, LVX, MEC, IMP, CFP/SBT, and SBT (Table S3 in the **Online Supplementary Document**)) demonstrated high potential clinical utility, with each being reported in susceptibility testing ≥10 times and with susceptibility frequencies at least five-fold greater than resistance frequencies (**Figure 3**, Panel A). We also noted serovar-specific therapeutic preferences: AMC demonstrated preferential use against *choleraesuis* infections, while GEN and AMK were predominantly employed for *typhimurium* cases. Notably, CRO exhibited reduced utilisation in *typhimurium* management. AMP maintained a broad-spectrum application across all serovars (**Figure 3**, Panel B).

## DISCUSSION

Our study provides the most extensive and up-to-date analysis of iNTS infection data. The statistical approaches and visualisations allow us to understand the past and present epidemiological patterns of iNTS infections in China and compare them with global epidemiological trends and disease burdens. Our results indicate that the geographic distribution of reported iNTS cases in China is not uniform, with the Central and Eastern regions seeing the most reported cases, which is consistent with the distribution of the country’s population. The temporal variations in case numbers across different areas are also noteworthy, with the number of reported cases in the Eastern region over the past 20 years far exceeding that of other regions. It remains to be determined whether this trend reflects a higher infection risk in the Eastern region or if it is related to some economic or healthcare-related factors.

Regarding differences in iNTS infection risk due to individual patient characteristics, we found that newborns under one year of age to be a high-risk group for iNTS infections, as they accounted for a significantly larger proportion of cases than any other age group. This may be partially explained by the greater likelihood of hospitalisation and blood culture testing among infants, as well as their relatively immature immune systems and increased behavioural exposure (*e.g.* mouthing objects) increasing their susceptibility to pathogens [53]. Similar trends have been observed in a previous infectious disease study [54], but more targeted research is required to delineate the specific drivers in the context of iNTS.

The notable predominance of males in iNTS cases observed here aligns with trends noted in research globally [15,55–58]. It remains unclear whether the observed gender disparity is biologically or socially mediated, as several mechanisms may be involved. Differences in sex hormones and gut microbiota between males and females may influence immune system function, potentially contributing to sex-specific patterns observed in various infectious diseases [59,60]. These biological pathways, however, require further investigation. In addition, socioeconomic factors such as occupational roles and lifestyle differences may also affect infection risk. For example, infections caused by the pig-associated serovar Choleraesuis have been linked to occupational or environmental exposures in pig farming and slaughtering environments [61]. According to national-level data from the China Statistical Yearbook 2023 [62], there is no substantial difference between male and female patients in hospital care utilisation, suggesting that disparities are unlikely to be solely attributable to differential health care access. This association, however, still warrants deeper research.

Studies from Africa noted that immunosuppression caused by HIV or malaria infections is a significant factor in the vulnerability to iNTS [25,63]. However, the proportion of iNTS patients co-infected with HIV in our study was very low, while China was certified malaria-free by the WHO on 30 June 2021 [64]. The most common risk factors in our sample were trauma or surgery, such as iNTS infections following craniocerebral trauma or caesarean section, suggesting that bacterial invasion resulting from disruptions of normal anatomical protective barriers, such as the skin, mucosal surfaces, or the blood-brain barrier in cases of cranio-cerebral trauma, may be a primary factor leading to iNTS infections. Furthermore, China is one of the countries with the heaviest burden of diabetes in the world [65], and our data show that patients with underlying conditions such as diabetes mellitus (type not specified in many reports) also account for a significant proportion of iNTS cases, indicating that the increased infection risk brought by underlying diseases is a considerable burden. Additionally, we have noted a higher proportion of cholecystitis and gallstones among iNTS patients. Recent studies have found that *Salmonella* can persist in bile and form biofilms, which may be an essential mechanism mediating iNTS infections [66].

Several limitations to our research must be considered. First, the included studies vary in methodology, sample size, and geography, limiting the generalisability of our conclusions. Moreover, case reports and small retrospective series may overrepresent severe or unusual iNTS cases, introducing potential publication and reporting bias. Such bias could affect estimates of case fatality rates, seroprevalence, and associated risk factors. Our review process also had certain limitations. For instance, the selection of studies was dependent on predefined inclusion and exclusion criteria, which may have led to the exclusion of relevant studies or resulted in the overrepresentation of certain types of research. Another limitation stems from the long temporal span (over 40 years) of the included literature. This meant that, although we inferred that most laboratories followed widely adopted domestic reference standards for common *Salmonella* serovars (such as the Chinese National Clinical Laboratory Guidelines), we encountered an absence of standardised reporting across periods, such as inconsistencies in the reported serovar/serogroup nomenclature and the laboratory identification methods used across studies. While we verified that all serovars reported in older studies remain valid under the current 9th edition of the White-Kauffmann-Le Minor scheme, full antigenic formulae were still missing in some cases. Such methodological variations cannot be fully reconciled, which may have influenced the comparability of serovar-specific analyses.

Lastly, our mortality analysis was based on unadjusted aggregate data, as individual-level information (*e.g.* age, underlying conditions, immune status, or definitive antimicrobial therapy) was not consistently available across studies. Consequently, we could not account for potential confounding effects in the reported subgroup differences. Readers should interpret the observed mortality trends with caution, as these may reflect differences in host characteristics, healthcare access, or reporting practices, rather than actual biological variation.

The results derived from our analysis may be somewhat discrepant from the actual situation of iNTS infections. Economic development and healthcare detection capabilities can affect the adequacy of case reporting, and there may be a lag between the reporting time and the actual occurrence of the cases. However, we had to rely on data from published literature when determining the status of iNTS infections, as national-level systematic surveillance, while more extensive, is not fully transparent or accessible to researchers who need it. Despite these limitations, our study still provides substantial insights into the prevalence of iNTS in China and advances our understanding of the potential hazards posed by *Salmonella* that has long existed between humans and animals.

## CONCLUSIONS

Based on 199 publications and 576 reported cases of iNTS in China, we observed distinct temporal and spatial trends, demographic patterns, pathogen diversity, and treatment practices. First, iNTS cases were concentrated in central and eastern regions, and were predominantly reported within tertiary hospitals, likely reflecting both referral bias and stronger surveillance capacity. Infants emerged as a key high-risk group, while trauma, surgery, and chronic diseases such as diabetes were major comorbid risk factors. Furthermore, Typhimurium, Choleraesuis, and Enteritidis remain the leading serovars, though their relative contributions to the case burden have shifted over time. Overall, the mortality rate of iNTS reported in China is 8.6% (95% CI = 6.8–10.4), lower than the global average of 14.5% (95% CI = 9.2–21.1), and the mortality rate has continuously declined in recent years. Antimicrobial resistance data suggest continued clinical utility of cephalosporins and fluoroquinolones, though resistance to certain agents is evident. 

Our findings have several implications. For policymakers, they highlight a need to establish standardised, national-level surveillance for iNTS in China. For researchers, it shows a need to prioritise prospective cohort studies, genomic surveillance to track invasive evolution, and evaluations of host-pathogen interactions in high-risk populations. In general, we note a need for targeted interventions that should address vulnerable groups such as neonates and patients with underlying conditions, as well as for strengthening antimicrobial stewardship and developing evidence-based treatment guidelines. In sum, iNTS remains a critical public health challenge requiring coordinated national and international strategies.

## Additional material


Online Supplementary Document
