# Supplementary material for: Invasive non-typhoidal Salmonella infections in China (1961–2024): a retrospective systematic analysis of multicentre case reports
Source: J Glob Health. 2026 Jan 12;16:04008. doi: 10.7189/jogh.16.04008 (PMC12796866; doi:10.7189/jogh.16.04008)
Supplement: Online Supplementary Document [file jogh-16-04008-s001.zip › jogh-16-04008-s001.pdf]

Supplement to: Haiyang Z, Chenghao J, Qianzhe C, Linlin H, Lin T, Zining W, Chenghu H, Fang H, Yan L, Guoping Z, Min Y. Invasive non-typhoidal Salmonella infections in China (1961 − 2024): a retrospective systematic analysis of multicentre case reports. J Glob Health. 2026;16:04008.

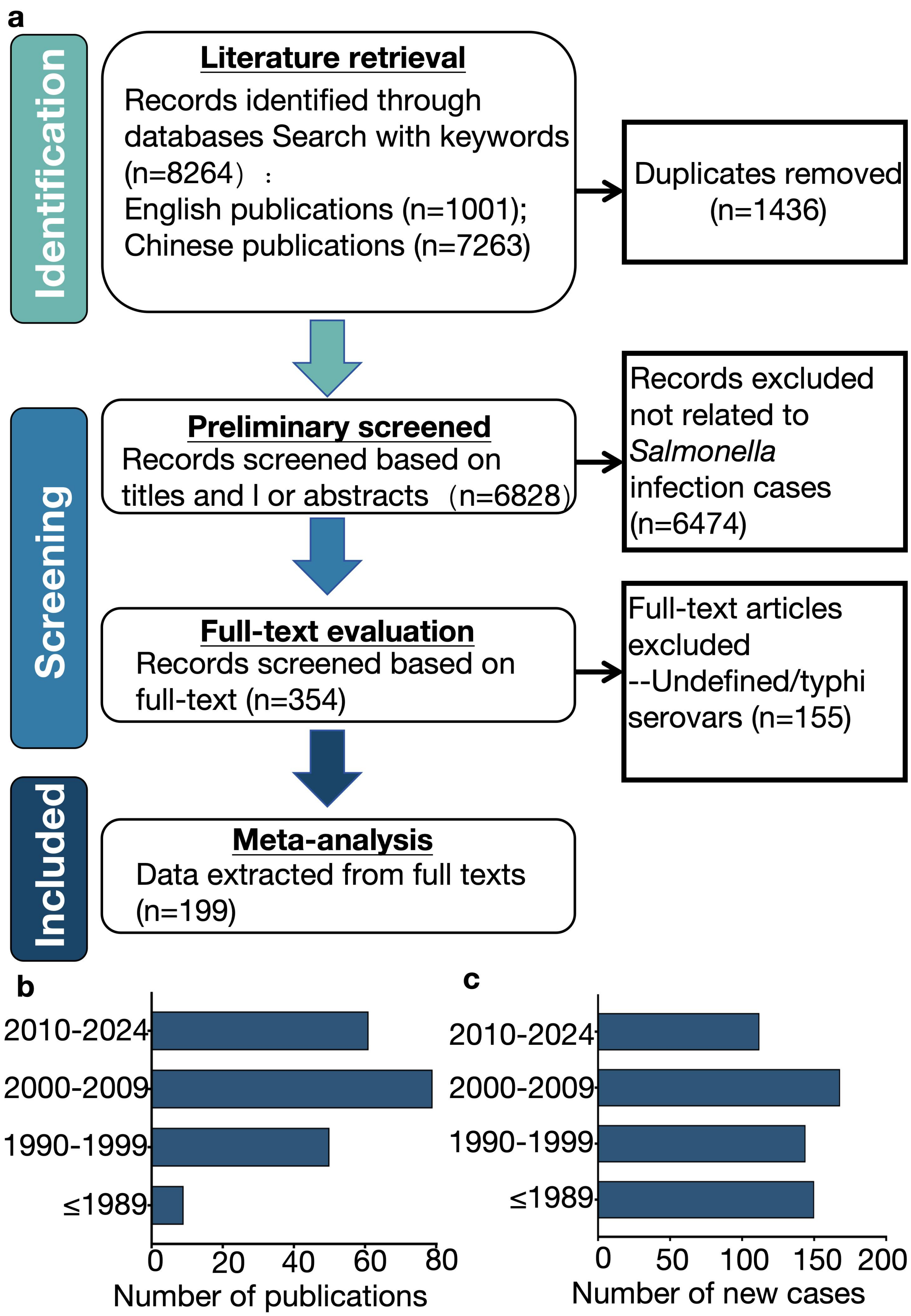

Figure S1. Analytic process and characteristics of the systematic review data set. **Panel A.** Search strategy and selection of articles registered on PROSPERO. **Panel B.** Number of publications included in the systematic review data set by year period . **Panel C.** Number of new cases recorded in publications included in the systematic review data set by year period.

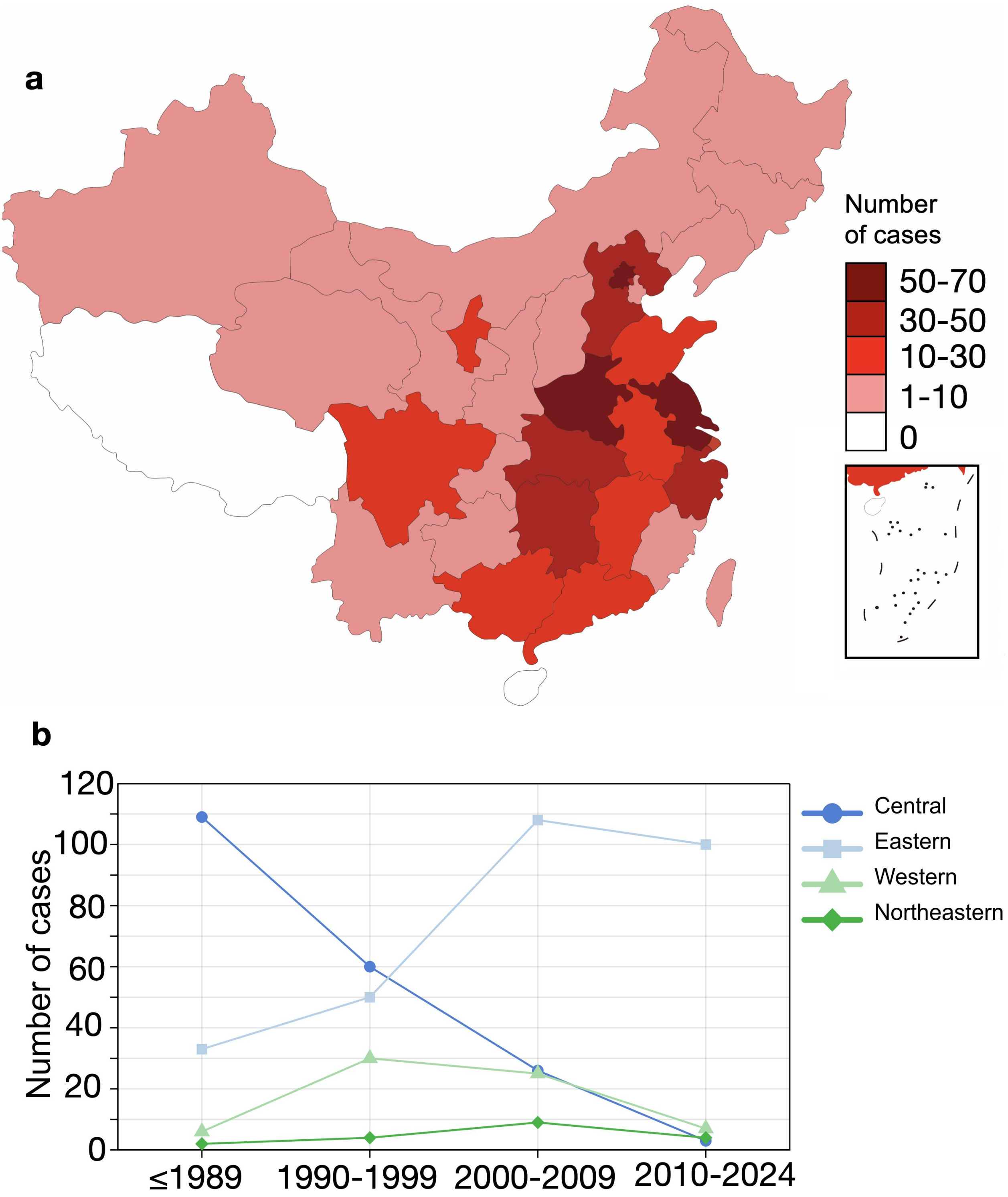

Figure S2. The spatiotemporal trend of iNTS in China. **Panel A.** In the data set, the number of reported iNTS infection cases varies by province. The colour intensity represents the number of cases, with darker shades indicating higher case numbers, while blank areas indicate regions where no iNTS infection cases were reported or data was not collected. **Panel B.** Trends in reported cases over time in the central, eastern, western, and northeastern economic regions.

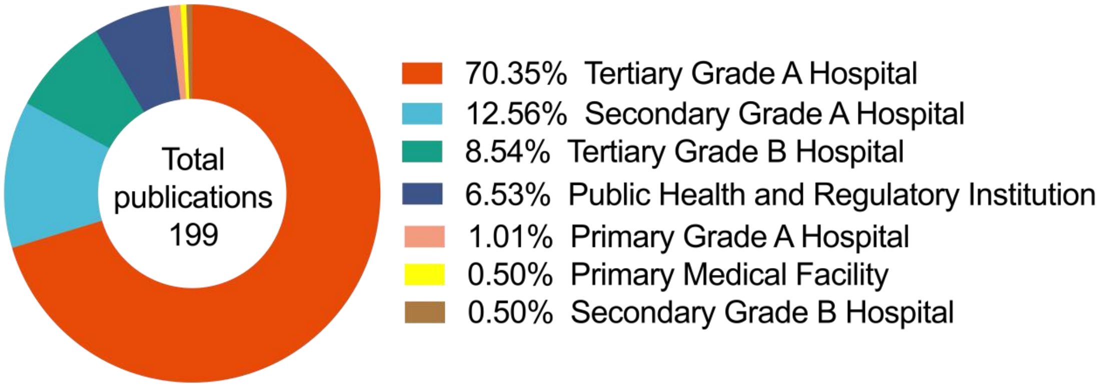

Figure S3. Classification of patient source institutions or primary author affiliations in all 199 included studies. The organizations were classified into seven categories: Tertiary Grade A Hospital, Secondary Grade A Hospital, Tertiary Grade B Hospital, Secondary Grade B Hospital, Primary Grade A Hospital, Primary Medical Facility, and Public Health and Regulatory Institution. This classification is based on the hospital's capacity for medical service provision.

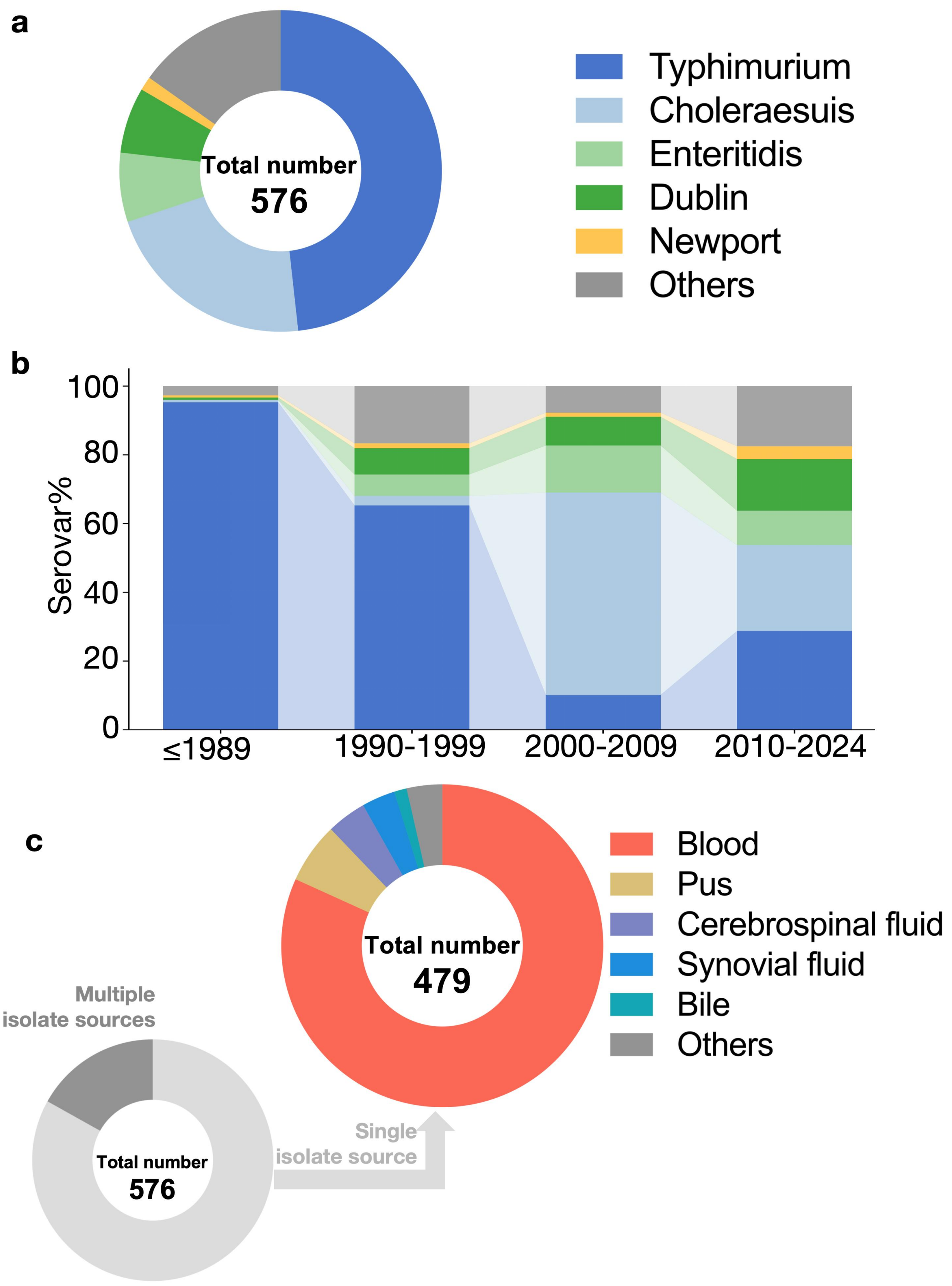

Figure S4. Etiological characteristics of iNTS infections. **Panel A.** Pie chart of the Top five serovars. **Panel B.** The proportion of the top five serovars across different time periods. **Panel C.** The sources of clinical isolates obtained from cultures.
